# 277. Low Rates of Bacterial Co-infection in Hospitalized Patients with COVID-19

**DOI:** 10.1093/ofid/ofab466.479

**Published:** 2021-12-04

**Authors:** Stephanie Spivack, Geena Kludjian, Stefania Gallucci, Laurie Kilpatrick, Aaron D Mishkin, Umadevi Sajjan, Vincent Tam, Jason C Gallagher, Jason C Gallagher

**Affiliations:** 1 Temple University Hospital, Philadelphia, Pennsylvania; 2 Temple University School of Pharmacy, Philadelphia, Pennsylvania; 3 Temple University, Philadelphia, Pennsylvania; 4 Temple University School of Medicine, Philadelphia, Pennsylvania; 5 Lewis Katz School of Medicine at Temple University, Philadelphia, Pennsylvania

## Abstract

**Background:**

The rate of bacterial co-infection in inpatients with COVID-19 is unknown, however, patients who are hospitalized with COVID-19 often receive antibiotics for community-acquired bacterial pneumonia (CABP). Reducing unnecessary antibiotic usage in this population is important to prevent adverse effects and slow the development of antimicrobial resistance.

**Methods:**

We performed a retrospective chart review on patients admitted to our health system between March and May 2020 with confirmed COVID-19 by nasopharyngeal PCR. We reviewed patients with positive cultures from urine, blood, sputum, and sterile sites. Positive cultures were reviewed to determine if they represented a true infection versus a contaminant or colonization. Patients with true infections were categorized as having a co-infection (CI) if the positive culture was collected within 48 hours of initial positive SARS-CoV-2 PCR test. Additional data was collected on patient demographics, types of infections, organisms grown, and antibiotic usage.

**Results:**

902 patients were admitted with positive SARS-CoV-2 tests during the study period. Of these, 47 patients (5.2%) had a bacterial CI. Some patients had more than one CI, with 53 total CIs identified. The median age of patients with CI was 66 years old (39 – 90). Tables 1 and 2 describe patient characteristics and infections. A subgroup analysis on types of bacteria was done on the 20 patients with a respiratory CI, who accounted for 2.2% of all COVID-positive patients admitted during the study period. In these infections, *Staphylococcus aureus, Streptococcus species,* and *Haemophilus influenzae* were the most common organisms, accounting for 60%, 15%, and 10% infections, respectively.

Table 1. Patient Characteristics

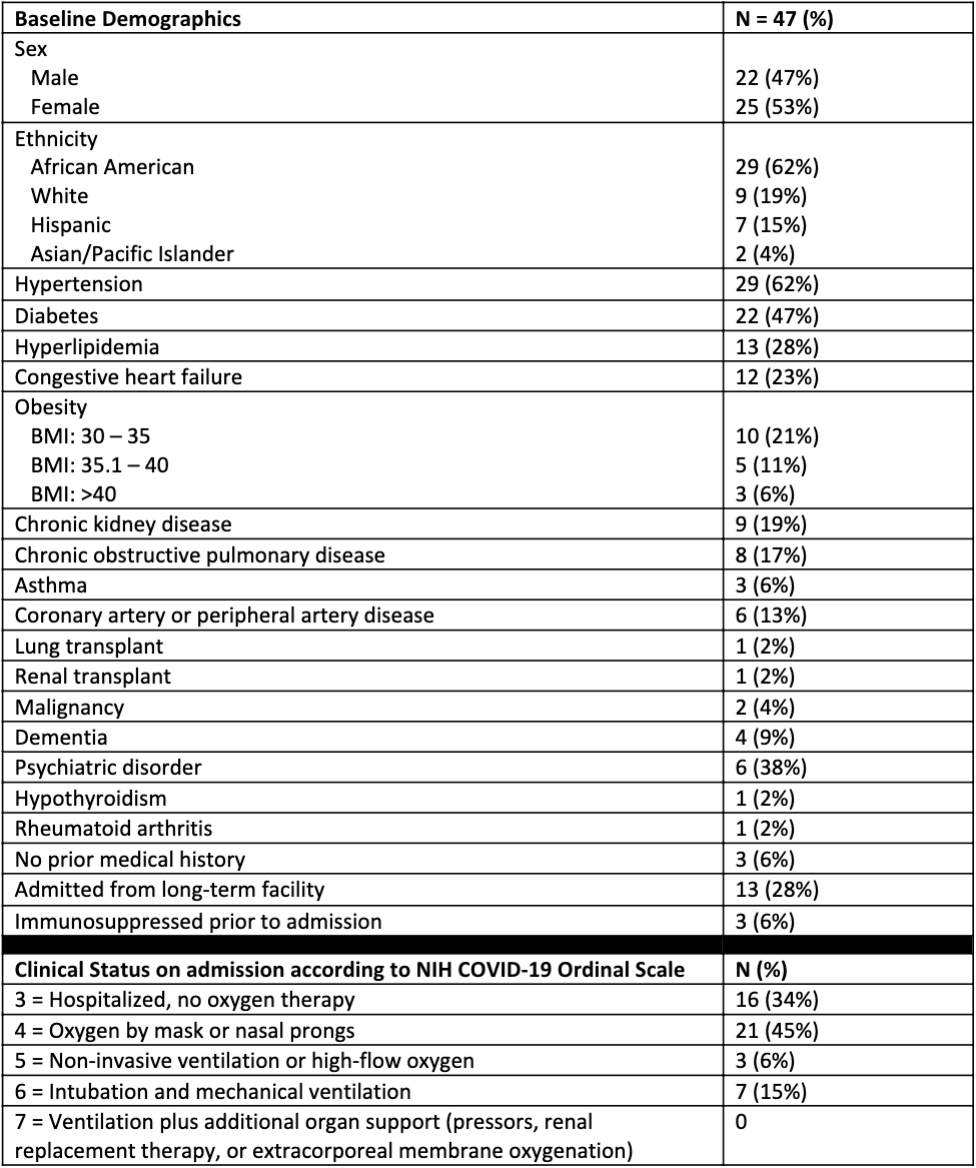

Table 2. Co-infections

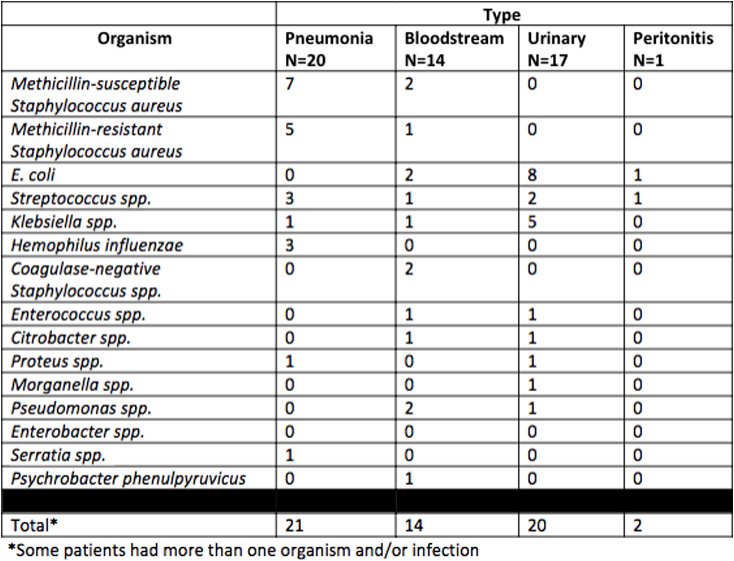

**Conclusion:**

The overall rate of CIs in patients admitted with COVID-19 was low. Some of these CIs may represent an “incidentally positive” COVID-19 test if a patient presented with one infection and had asymptomatic carriage of SARS-CoV-2 when community prevalence was high. Further analysis is needed to evaluate specific risk factors for co-infection.

**Disclosures:**

**Jason C. Gallagher, PharmD, FIDP, FCCP, FIDSA, BCPS**, **Astellas** (Consultant, Speaker’s Bureau)**Merck** (Consultant, Grant/Research Support, Speaker’s Bureau)**Qpex** (Consultant)**scPharmaceuticals** (Consultant)**Shionogi** (Consultant) **Jason C. Gallagher, PharmD, FIDP, FCCP, FIDSA, BCPS**, Astellas (Individual(s) Involved: Self): Speakers' bureau; Merck (Individual(s) Involved: Self): Consultant, Grant/Research Support; Nabriva: Consultant; Qpex (Individual(s) Involved: Self): Consultant; Shionogi (Individual(s) Involved: Self): Consultant

